# Evaluation of Textbook Outcome as a Composite Quality Measure of Elective Laparoscopic Cholecystectomy

**DOI:** 10.1001/jamanetworkopen.2022.32171

**Published:** 2022-09-20

**Authors:** James Lucocq, John Scollay, Pradeep Patil

**Affiliations:** 1 Department of General and Upper GI Surgery, Ninewells Hospital, Dundee, United Kingdom

## Abstract

**Importance:**

A textbook outcome (TO) is a composite quality measure that incorporates multiple perioperative events to reflect the most desirable outcome. The use of TO increases the event rate, captures more outcomes to reflect patient experience, and can be used as a benchmark for quality improvement.

**Objectives:**

To introduce the concept of TO to elective laparoscopic cholecystectomy (LC), propose the TO criteria, and identify characteristics associated with TO failure.

**Design, Setting, and Participants:**

This retrospective cohort study was performed at 3 surgical units in a single health board in the United Kingdom. Participants included all patients undergoing elective LC between January 1, 2015, and January 1, 2020. Data were analyzed from January 1, 2015, to January 1, 2020.

**Main Outcomes and Measures:**

The TO criteria were defined based on review of existing TO metrics in the literature for other surgical procedures. A TO was defined as an unremarkable elective LC without conversion to open cholecystectomy, subtotal cholecystectomy, intraoperative complication, postoperative complications (Clavien-Dindo classification ≥2), postoperative imaging, postoperative intervention, prolonged length of stay (>2 days), readmission within 100 days, or mortality. The rate of TOs was reported. Reasons for TO failure were reported, and preoperative characteristics were compared between TO and TO failure groups using both univariate analysis and multivariable logistic regressions.

**Results:**

A total of 2166 patients underwent elective LC (median age, 54 [range, 13-92] years; 1579 [72.9%] female). One thousand eight hundred fifty-one patients (85.5%) achieved a TO with an unremarkable perioperative course. Reasons for TO failure (315 patients [14.5%]) included conversion to open procedure (25 [7.9%]), subtotal cholecystectomy (59 [18.7%]), intraoperative complications (40 [12.7%]), postoperative complications (Clavien-Dindo classification ≥2; 92 [29.2%]), postoperative imaging (182 [57.8%]), postoperative intervention (57 [18.1%]), prolonged length of stay (>2 days; 142 [45.1%]), readmission (130 [41.3%]), and mortality (1 [0.3%]). Variables associated with TO failure included increasing American Society of Anesthesiologists score (odds ratio [OR], 2.55 [95 CI, 1.69-3.85]; *P* < .001), increasing number of prior biliary-related admissions (OR, 2.68 [95% CI, 1.36-5.27]; *P* = .004), acute cholecystitis (OR, 1.42 [95% CI, 1.08-1.85]; *P* = .01), preoperative endoscopic retrograde cholangiopancreatography (OR, 2.07 [95% CI, 1.46-2.92]; *P* < .001), and preoperative cholecystostomy (OR, 3.22 [95% CI, 1.54-6.76]; *P* = .002).

**Conclusions and Relevance:**

These findings suggest that applying the concept of TO to elective LC provides a benchmark to identify suboptimal patterns of care and enables institutions to identify strategies for quality improvement.

## Key Points

**Question:**

Can the concept of textbook outcome (TO) be applied to elective laparoscopic cholecystectomy (LC), and if so, what are the TO criteria and the characteristics associated with TO failure?

**Findings:**

In this cohort study of 2166 participants undergoing elective LC, 1851 (85.5%) achieved a TO with an unremarkable perioperative course. Predisposing factors and those contributing to TO failure were identified.

**Meaning:**

These findings suggest that applying the concept of TO to elective LC provides a benchmark to enable institutions to identify strategies for quality improvement.

## Teaser

This cohort study proposes the textbook outcome criteria after elective cholecystectomy and identifies reasons for failure and individual patient factors predisposing to failure.

## Introduction

Textbook outcome (TO) is a composite quality measure that incorporates multiple perioperative outcomes to reflect the most desirable outcome. It is a multidimensional indicator that considers many aspects of morbidity (eg, complications, prolonged admission, further intervention, and readmission) and incorporates multiple facets of patient care. Reliance on single outcomes with low event rates does not reflect the perioperative course accurately and does not incite improvements in quality of care.^1,2,3,4^

To date a TO has not been described after elective LC. Although bile duct injury is the most emphasized complication, it is infrequent and does not capture the extent of perioperative morbidity when used as an outcome measure. Even referring to overall complication rates (eg, through the Clavien-Dindo classification) will fail to acknowledge the extent of postoperative problems such as prolonged length of stay and readmission.

Reporting a TO will acknowledge all adverse perioperative outcomes, giving a better impression of quality of care as a metric of assessment.^5,6,7,8,9,10,11^ A TO metric will provide a holistic perspective on outcomes better aligned with patient experiences and improve transparency of reporting vs single-outcome metrics. It may also highlight variation in outcomes between centers and provide a benchmark standard of care.^12,13,14,15,16,17^ The aims of this cohort study were to propose the TO criteria after elective LC and to identify reasons for TO failure and individual patient factors predisposing to failure.

## Methods

### Population Cohort

All patients who underwent elective LC in 1 health board in the United Kingdom (consisting of 1 tertiary referral center and 2 satellite units) between January 1, 2015, and January 1, 2020, were included in this retrospective cohort study. The health board covers a defined geographical region with a stable population of approximately 493 000 people. Indications for elective LC included all symptomatic biliary pathology (eg, biliary colic, cholecystitis, gallstone pancreatitis). Ethical approval was granted by the Caldicott Guardian and the regional information governance service, which waived the need for informed consent for deidentified retrospective data. This study follows the Strengthening the Reporting of Observational Studies in Epidemiology (STROBE) reporting guideline.

**Figure 1.**
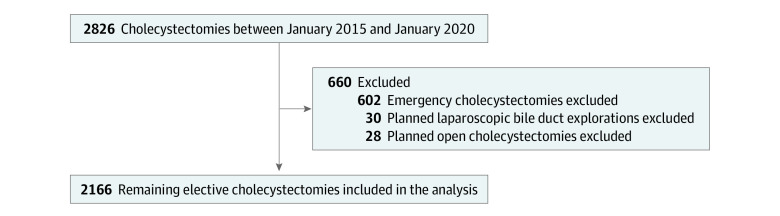
Study Design

Emergency cholecystectomies, which were excluded, were defined as any cholecystectomy performed during an acute admission, whereas elective cholecystectomies were strictly those performed on an outpatient basis, whether or not they were performed as an interval procedure. Planned open cholecystectomies and planned bile duct explorations were excluded in the analysis because both are likely to have a different expected perioperative course (Figure 1).

### Data Collection

Data were collected retrospectively from multiple databases using a deterministic records-linkage method. Patients were tracked between databases using a unique 10-digit patient identifier. These databases were used to obtain information relating to both previous gallstone-related admissions and the operative admission and included baseline demographic and operative data. Details of any perioperative adverse outcomes (eg, complications, imaging, or intervention) were recorded. Total length of stay (LOS) was recorded for all patients. Records of those patients who were readmitted under surgical care within 100 days of their operation were scrutinized for details of any further complications, imaging, or intervention.

### Defining TO

The literature was reviewed and TO was defined as an elective LC performed in the absence of conversion to open cholecystectomy, subtotal cholecystectomy, intraoperative complication, postoperative complication (Clavien-Dindo classification ≥2), postoperative imaging or intervention, prolonged postoperative LOS, readmission, or mortality. An LOS greater than 2 postoperative days was regarded as prolonged because this LOS was associated with the other perioperative adverse outcomes.

### Reasons for TO Failure and Predisposing Factors

The overall rate of TO was calculated, and the perioperative outcomes contributing to failure were reported. The associations between adverse perioperative outcomes were found to identify interconnected aspects of these outcomes. By determining these associations, surgical units can begin to identify suboptimal patterns of care.

### Statistical Analysis

Data were analyzed from January 1, 2015, to January 1, 2020. Factors associated with achieving TO were determined using univariate and multivariable analysis. Factors included in the analysis included age (<40, 40-59, and ≥60 years), American Society of Anesthesiologists (ASA) score (1, 2, or ≥3), diagnosis (biliary colic, cholecystitis, biliary pancreatitis), radiological findings (wall thickening [≥4 mm], pericholecystic fluid), number of previous biliary admissions and preoperative interventions (endoscopic retrograde cholangiopancreatography [ERCP], cholecystostomy). In the multivariable logistic regression, a top-down approach was used, eliminating the most insignificant variable until significant variables remain. Multicollinearity between all variables were determined using Pearson correlation coefficients (*r* values), and collinearity coefficients greater than 0.25 were reported. All statistical tests were conducted using the STATA IC, version 16.1, statistical software package (StataCorp LLC). One-sided *P* < .05 was considered statistically significant.

## Results

### Background

During the 5-year period, 2166 elective cholecystectomies were performed. The median age of the cohort was 54 (range, 13-92) years. The cohort was predominantly female (1579 [72.9%] vs 587 [27.1%] male), and the median ASA score was 2 (range, 1-4) (Table 1).

**Table 1.** Preoperative Univariate Comparison Between TO and TO-Failure Groups

Characteristic	Patient group^a^			*P* value
	All (N = 2166)	TO achieved (n = 1851)	TO not achieved (n = 315)	
Age, median (range), y	54 (13-92)	53 (13-86)	58 (18-91)	<.001
Sex, %				
Male	587 (27.1)	474 (25.6)	113 (35.9)	<.001
Female	1579 (72.9)	1377 (74.4)	202 (64.1)	
ASA score				
1	726 (33.5)	656 (35.4)	70 (22.2)	<.001
2	1227 (56.6)	1036 (56.0)	191 (60.6)	
≥3	213 (9.8)	159 (8.6)	54 (17.1)	
Indication				
Biliary colic	1262 (58.3)	1133 (61.2)	129 (41.0)	<.001
Cholecystitis	703 (32.4)	557 (30.1)	146 (46.3)	<.001
Gallstone pancreatitis	91 (4.2)	76 (4.1)	15 (4.8)	.29
Other (eg, biliary dyskinesia, polyps)	110 (5.1)	85 (4.6)	25 (7.9)	.01
Imaging				
US of abdomen	2086 (96.3)	1792 (96.8)	294 (93.3)	.003
MRCP	647 (29.9)	516 (27.9)	131 (41.6)	<.001
CT of abdomen and/or pelvis	306 (14.1)	216 (11.7)	90 (28.6)	<.001
Preoperative radiological findings				
Thickened gallbladder wall	666 (30.7)	528 (28.5)	138 (43.8)	<.001
Pericholecystic fluid	276 (12.7)	223 (12.0)	53 (16.8)	.02
Common bile duct stones	158 (7.3)	101 (5.5)	57 (18.1)	<.001
Preoperative ERCP	178 (8.2)	110 (5.9)	68 (21.6)	<.001
Preoperative cholecystostomy	32 (1.5)	13 (0.7)	19 (6.0)	<.001
No. of previous biliary-related admissions				
1	762 (35.2)	637 (34.4)	125 (39.7)	<.001
2	139 (6.4)	97 (5.2)	42 (13.3)	
≥3	50 (2.3)	33 (1.8)	17 (5.4)	
Previous abandoned cholecystectomy	19 (0.9)	9 (0.5)	10 (3.2)	<.001

Abbreviations: ASA, American Society of Anesthesiologists; CT, computed tomography; ERCP, endoscopic retrograde cholangiopancreatography; MRCP, magnetic resonance cholangiopancreatography; TO, textbook outcome; US, ultrasonography.

^a^ Unless otherwise indicated, data are expressed as No. (%) of patients. Percentages have been rounded and may not total 100.

Overall, 4 patients had bile duct injuries (0.2%), only 1 of which was a complete transection of the bile duct (0.05%). In the entire study cohort, postoperative intervention was required in 57 patients and included ERCP (n = 30), laparoscopy (n = 15), laparotomy (n = 10), and radiologically guided drainage (n = 5). The indications for return to operating theater were collections (n = 9), bile leak (n = 8), bowel perforations (n = 3), hemorrhage (n = 2), bowel obstruction (n = 2), and oncologic resection secondary to gallbladder cancer (n = 1). The radiologically guided drainages were performed for intra-abdominal collections (n = 5).

One hundred eighty-two patients underwent postoperative imaging, of whom 178 (97.8%) had symptoms or worsening clinical signs. One hundred thirty patients (6.0%) were readmitted during the 100-day follow-up period (total of 158 admissions; median time from admission, 9 [range, 0-97] days; median length of admission, 2 [range, 1-70] days). The most common causes for readmission included collections, retained stone, and wound infections. One patient died during the 100-day follow-up period (morality rate, 0.05%) owing to unexpected disseminated malignant disease.

### TO and Its Contributors

**Figure 2.**
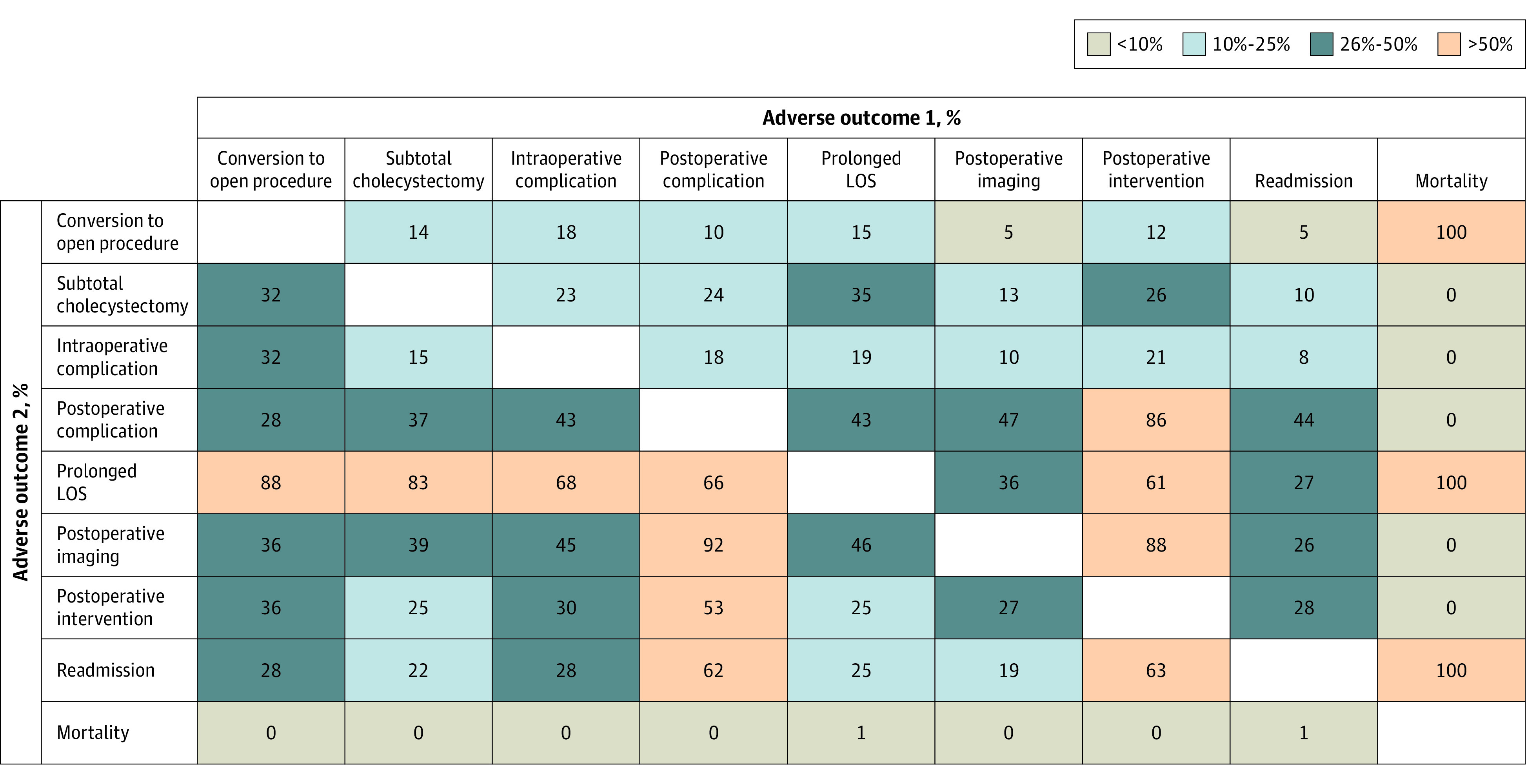
Proportion of Patients With Adverse Outcome 2 Among Those With Adverse Outcome 1 LOS indicates length of stay.

A total of 1851 patients (85.5%) had a TO for elective LC with an unremarkable perioperative and postoperative course without subtotal cholecystecomy, conversion to open cholecystecomy, complication, prolonged LOS, postoperative imaging or intervention, readmission, or mortality (Table 1). Perioperative outcomes and reasons for TO failure (315 [14.5%]) are reported in Table 2. As demonstrated, the most frequent contributors toward TO failure were postoperative imaging (59 [57.8%]), prolonged LOS (142 [45.1%]), readmission (130 [41.3%]), and postoperative complication (92 [29.2%]). The associations between adverse outcomes are demonstrated in Figure 2 to identify interconnected aspects of outcomes and further interpret quality of care.

**Table 2.** Perioperative Data for Patients Undergoing Elective Laparoscopic Cholecystectomy

Outcome	No. (%) of patients		
	All (N = 2166)	Contribution to TO failure (n = 315)	Single contributor to TO (n = 315)
Converted to open cholecystectomy	25 (1.2)	25 (7.9)	4 (1.3)
Subtotal cholecystectomy	59 (2.7)	59 (18.7)	8 (2.5)
Intraoperative complications	40 (1.8)	40 (12.7)	12 (3.8)
Postoperative complications (Clavien-Dindo classification ≥2)	92 (4.2)	92 (29.2)	4 (1.3)
Postoperative imaging	182 (8.4)	182 (57.8)	46 (14.6)
Postoperative intervention	57 (2.6)	57 (18.1)	0
Prolonged LOS (>2 d)	142 (6.5)	142 (45.1)	31 (9.8)
Readmission	130 (6.0)	130 (41.3)	23 (7.3)
Mortality	1 (0.05)	1 (0.3)	0

Abbreviations: LOS, length of stay; TO, textbook outcome.

### Risk Factors for TO Failure

In the univariate analysis, patients not achieving TO were more likely to be older (median age, 58 [range, 18-91] vs 53 [range, 13-86] years), to be male (113 of 315 [35.9%] vs 474 of 1851 [25.6%]), to have an ASA score of at least 2 (245 of 315 [77.8%] vs 1195 of 1851 [64.5%]), and to have had cholecystitis (146 of 315 [46.3%] vs 538 of 1851 [29.1%]) (Table 1). Patients not achieving TO also were more likely to have had at least 2 previous biliary-related admissions (59 of 315 [18.7%] vs 130 of 1851 [7.0%]) and to have undergone a preoperative ERCP (68 of 315 [21.6%] vs 110 of 1851 [5.9%]), cholecystostomy (19 of 315 [6.0%] vs 13 of 1851 [0.7%]), or previous abandoned elective LC (10 of 315 [3.2%] vs 9 of 1851 [0.5%]) (Table 1).

In the multivariable analysis, the following variables were positively associated with failure to achieve TO: ASA score of at least 2 (odds ratio [OR], 1.57 [95% CI, 1.17-2.12]; *P* = .003), at least 2 previous admissions (OR, 1.80 [95% CI, 1.18-2.76]; *P* = .007), cholecystitis (OR, 1.42 [95% CI, 1.08-1.85]; *P* = .01), preoperative ERCP (OR, 2.07 [95% CI, 1.46-2.92]; *P* < .001), and cholecystostomy (OR, 3.22 [95% CI, 1.54-6.76]; *P* = .002) (Table 3). Of all variables considered for inclusion in the final model, we identified significant collinearity (*r* ≥ 0.25) between previous cholecystitis and both thickened wall (*r* = 0.81) and pericholecystic fluid (*r* = 0.51).

**Table 3.** Multivariable Logistic Regression of Variables Associated With Not Achieving TO

Independent variable for outcome of TO failure	OR (95% CI) [SE]	Z	*P* value
ASA score			
2	1.57 (1.17-2.12) [0.24]	2.99	.003
≥3	2.55 (1.69-3.85) [0.53]	4.49	<.001
No. of previous admissions			
2	1.80 (1.18-2.76) [0.39]	2.72	.007
≥3	2.68 (1.36-5.27) [0.93]	2.85	.004
Cholecystitis	1.42 (1.08-1.85) [0.19]	2.55	.01
ERCP	2.07 (1.46-2.92) [0.37]	4.12	<.001
Cholecystostomy	3.22 (1.54-6.76) [1.21]	3.10	.002

Abbreviations: ASA, American Society of Anesthesiologists; ERCP, endoscopic retrograde cholangiopancreatography; OR, odds ratio; TO, textbook outcome.

## Discussion

The concept of the TO was first described by Kolfschoten et al,^18^ who reported this composite outcome measure in patients undergoing colonic resection for cancer. However, this approach has since been extended to noncancer surgery (aortic aneurysm repair, liver transplant).^19,20^ Definitions of TO typically include freedom from perioperative morbidity, early mortality, readmission, and procedure-specific variables such as margin status and lymph node yield.^18,20,21,22,23,24,25,26^ Single complication indicators such as bile duct injuries after LC have a low event rate and therefore do not represent the multidimensional approach of the surgical process. Furthermore, acknowledging complication rates in general will not reflect all the perioperative problems that a patient may face. The use of TO increases the event rate and captures more outcomes to reflect patient experience. To our knowledge, this study is the first to apply TO to elective LC, propose the TO criteria as a performance assessment tool, and report risk factors for TO failure.

The proposed TO criteria is a perioperative course not affected by conversion to open cholecystectomy, subtotal cholecystectomy, intraoperative complication, postoperative complication (Clavien-Dindo classification ≥2), postoperative imaging or intervention, prolonged postoperative LOS (>2 days), readmission, or mortality. Textbook outcome was achieved in most patients (85.5%), and the most frequent contributors toward TO failure were postoperative imaging (57.8%), prolonged LOS (45.1%), readmission (41.3%), and postoperative complication (29.2%). Although TO rates have not been reported before, the rates of the reported adverse outcomes are broadly consistent with those reported by both the CholeS group and a single surgeon series.^27^

Processes of care represent the actual care delivered to a patient and play a fundamental role in hospital quality assessment. Measuring and reporting process measures can be facilitated by establishing a clear definition of the population or procedure being studied (denominator) and what represents a success or a failure (numerator). Finally, process measures can be used as clinical quality indictors to facilitate more proactive quality improvement. It has been argued that there must be a direct link to a defined outcome for a process measure to be valid, and as such, proposing TO helps to facilitate this process.^4,25^

Benchmarks are a fundamental part of the quality improvement process to which institutions refer and compare their outcomes. Setting benchmarks through the format of TO can provide an internationally recognized standard of care to improve assessment of individual hospital performance and assist with quality improvement activity. These benchmarks should continuously evolve over time to drive progressive quality improvement.^4,18,20^ As demonstrated, this study sets the criteria for this benchmark and should be regarded as a stepping stone to the establishment of internationally recognized benchmark rates.^20^ As the concept of TO becomes more widely disseminated, definitions proposed at the institutional level may motivate revision of data captured in databases to facilitate both validation and revision of this metric, and comparison of outcomes across institutions.

A significant strength of TO is its multidimensional perspective as a composite quality of care indicator. Just as TO criteria after oncologic surgery include multiple outcomes (eg, resection margins, nodal harvest, and survival rates), TO after elective LC incorporates all adverse outcomes that would deviate from an unremarkable outcome. This approach can identify various suboptimal patterns of perioperative care to guide quality improvement, which is not possible when depending on single infrequent outcomes.^20,24^ For example, in the present cohort, prolonged LOS and readmission were among the most frequent reasons for TO failure and interconnected aspects between outcomes are reported. Quality improvement processes to reduce these rates with reference to internationally accepted benchmarks would help improve outcomes.

It has been demonstrated that the patient consent process before LC is often inadequate. The concept of TO may be useful in helping obtain informed preoperative consent.^12,13,14,15,16,17^ Traditionally, precholecystectomy discussion has focused heavily on bile duct injury, which, given its potentially catastrophic consequences, will always remain a cornerstone of the consent process. Although individual complications may portend differential implications for long-term prognosis, understanding of the multitude of immediate outcomes may be particularly important to patients and their view of the health care experience.^23^ Reference to textbook outcome rates during a consultation provides a multidimensional account of the quality of the perioperative care that is better aligned with patient experience compared with any single metric.^1,2,3,21,22,28,29,30^ This study reports a significant proportion of patients who do not achieve TO (14.5%) and its contributors. Outlining the perioperative course in this way provides a more comprehensive and representative picture than quoting infrequent outcomes. Of course, acknowledging patient risk factors for TO failure will help surgeons appreciate patient variation and guide an individualized consent process. In the present study, an ASA score of at least 2, at least 2 previous biliary-related emergency admissions, previous cholecystitis, preoperative ERCP, and cholecystostomy were associated with TO failure.

Elective LC is an extremely common operation consuming large amounts of surgical and financial resources.^31^ Applying TO rates to cholecystectomy will also help guide those bodies responsible for funding and organizing health services. Many elective LCs will be straightforward procedures with no follow-up required. Determining that 14.5% of patients undergoing elective LC need ongoing surgical care will inform appropriate resource allocation and health care organization.

When considering factors to be included in this TO model, several potential options were appraised but ultimately rejected. Conventional wisdom would suggest that an intraoperative critical view of safety should be achieved during every elective LC. Although obtaining a critical view might be regarded as normal practice, there are other methods of safely performing a cholecystectomy. Indeed, some evidence suggests that a critical view is obtained much less than commonly assumed.^32^ Therefore we believed it was inappropriate to include this as a TO objective. Other variables related to the quality of surgery may be considered, such as prolonged operative time, intraoperative bile spillage, blood loss, or postoperative pain. Although these variables have been found to be associated with varied patient outcomes, it might be difficult to obtain accurate measurements, and these parameters might not necessarily imply lower surgical quality.^25^

Relief of biliary symptoms may initially appear a logical criterion to include; however, such symptoms can be variable, unpredictable, and subjective. Some patients may also develop new issues such as postcholecystectomy pain or diarrhea. Although developing such symptoms is troublesome, these are not markers of the quality of the operation; hence, they were not considered as TO criteria. These patient-related outcomes should be considered in a global outcome measure that assesses quality of life after elective LC.

### Limitations

The present study has limitations. First, the cohort is from a single health board and is unlikely to be representative of nationwide or international data. Nevertheless, the aim was not to set the internationally accepted benchmark rates, but instead to introduce the concept of TO for elective LC and to demonstrate the advantages of TO as a quality improvement metric. Multicenter cohort studies should be conducted to establish accepted TO benchmarks rates to drive quality improvement. Second, further limitation is the standardization of terms. Although most constituents of the TO criteria are self-explanatory, the definition of subtotal cholecystectomy is open to interpretation, and there are multiple variations in surgical technique. As such, accuracy of reporting of subtotal cholecystectomy is likely to vary more than the other outcome measures. Similarly, the accepted inclusion criteria may vary by expect opinion. For example, an expert may only consider Clavien-Dindo classification of at least 3 a severe enough complication to define TO failure vs Clavien-Dindo classification of at least 2. However, the criteria in this study have all been used in previous TO research after other operations and increase the credibility of the criteria used herein.^4^ One may argue that the application of TO to elective LC is not a worthy endeavor, given the high rate of TO achievement. However, the frequency of elective LC, particularly in Western populations, means that the procedure incurs great cost and results in high absolute morbidity, and thus the importance of maximizing TO is vital.^33,34,35,36^ Last, if TO would be implemented as a part of a nationwide audit, defensive strategies such as strict patient selection in certain centers could emerge. This emphasizes that TO should always be used in addition to the other quality indicators. Textbook outcomes are not designed to replace single-quality indicators, but as an additional tool. In this sense, patient-reported outcome measures will gain significance in future quality programs.^37^

## Conclusions

In this cohort study, we introduce the concept of TO after elective LC, propose the TO criteria, and identify reasons for TO failure. Our findings suggest that the use of TO increases the event rate, captures more aspects of patient care and experience, and provides a multidimensional view of patient outcomes. These outcomes should help better assessment of the quality of care provided and drive quality improvement.

## References

[1] National Institute for Health and Care Excellence. Single-incision laparoscopic cholecystectomy: interventional procedures guidance. December 17, 2014. Accessed March 30, 2022. https://www.nice.org.uk/guidance/ipg50

[2] National Institute for Health and Care Excellence. Gallstone disease: diagnosis and management. October 29, 2014. Accessed March 30, 2022. https://www.nice.org.uk/guidance/cg188 25473723

[3] Abbott TEF, Fowler AJ, Dobbs TD, Harrison EM, Gillies MA, Pearse RM. Frequency of surgical treatment and related hospital procedures in the UK: a national ecological study using hospital episode statistics. Br J Anaesth. 2017;119(2):249-257. doi:10.1093/bja/aex137 28854546

[4] Keus F, de Jong JA, Gooszen HG, van Laarhoven CJ. Laparoscopic versus open cholecystectomy for patients with symptomatic cholecystolithiasis. Cochrane Database Syst Rev. 2006;(4):CD006231. doi:10.1002/14651858.CD006231 17054285 PMC13378884

[5] Cuschieri A, Dubois F, Mouiel J, . The European experience with laparoscopic cholecystectomy. Am J Surg. 1991;161(3):385-387. doi:10.1016/0002-9610(91)90603-B 1825763

[6] Vecchio R, MacFadyen BV, Latteri S. Laparoscopic cholecystectomy: an analysis on 114 005 cases of United States series. Int Surg. 1998;83(3):215-219. 9870777

[7] Moore DE, Feurer ID, Holzman MD, . Long-term detrimental effect of bile duct injury on health-related quality of life. Arch Surg. 2004;139(5):476-481. doi:10.1001/archsurg.139.5.476 15136346

[8] van Boxel GI, Hart M, Kiszely A, Appleton S. Elective day-case laparoscopic cholecystectomy: a formal assessment of the need for outpatient follow-up. Ann R Coll Surg Engl. 2013;95(8):e142-e146. doi:10.1308/rcsann.2013.95.8.561 24165332 PMC4311530

[9] Down SK, Nicolic M, Abdulkarim H, Skelton N, Harris AH, Koak Y. Low ninety-day re-admission rates after emergency and elective laparoscopic cholecystectomy in a district general hospital. Ann R Coll Surg Engl. 2010;92(4):307-310. doi:10.1308/003588410X12664192075053 20385048 PMC3025213

[10] Mjåland O, Raeder J, Aasboe V, Trondsen E, Buanes T. Outpatient laparoscopic cholecystectomy. Br J Surg. 1997;84(7):958-961. doi:10.1002/bjs.1800840714 9240135

[11] Taylor E, Gaw F, Kennedy C. Outpatient laparoscopic cholecystectomy feasibility. J Laparoendosc Surg. 1996;6(2):73-77. doi:10.1089/lps.1996.6.73 8735042

[12] Lau H, Brooks DC. Contemporary outcomes of ambulatory laparoscopic cholecystectomy in a major teaching hospital. World J Surg. 2002;26(9):1117-1121. doi:10.1007/s00268-002-6264-1 12209240

[13] Busweiler LAD, Schouwenburg MG, van Berge Henegouwen MI, ; Dutch Upper Gastrointestinal Cancer Audit (DUCA) group. Textbook outcome as a composite measure in oesophagogastric cancer surgery. Br J Surg. 2017;104(6):742-750. doi:10.1002/bjs.10486 28240357

[14] Poelemeijer YQM, Marang-van de Mheen PJ, Wouters MWJM, Nienhuijs SW, Liem RSL. Textbook outcome: an ordered composite measure for quality of bariatric surgery. Obes Surg. 2019;29(4):1287-1294. doi:10.1007/s11695-018-03642-1 30569369

[15] van der Kaaij RT, de Rooij MV, van Coevorden F, . Using textbook outcome as a measure of quality of care in oesophagogastric cancer surgery. Br J Surg. 2018;105(5):561-569. doi:10.1002/bjs.10729 29465746

[16] General Medical Council. Decision making and consent. November 9, 2020. Accessed March 30, 2022. https://www.gmc-uk.org/ethical-guidance/ethical-guidance-for-doctors/decision-making-and-consent

[17] Courtney MJ, Royle TJ. Current use of procedure specific consent forms for laparoscopic cholecystectomy. Ann R Coll Surg Engl. 2018;100(6):446-449. doi:10.1308/rcsann.2018.0099 29962300 PMC6111902

[18] Kolfschoten NE, Kievit J, Gooiker GA, . Focusing on desired outcomes of care after colon cancer resections; hospital variations in “textbook outcome”. Eur J Surg Oncol. 2013;39(2):156-163. doi:10.1016/j.ejso.2012.10.007 23102705

[19] Uzzaman MM, Tayeh S, Sinha S, Ratnasingham K, Stoker DL. Consenting practice for laparoscopic cholecystectomy: are we doing enough to warn patients about their operation? Int J Surg. 2011;9(8):643-647. doi:10.1016/j.ijsu.2011.08.004 21945868

[20] Bottrell MM, Alpert H, Fischbach RL, Emanuel LL. Hospital informed consent for procedure forms: facilitating quality patient-physician interaction. Arch Surg. 2000;135(1):26-33. doi:10.1001/archsurg.135.1.26 10636343

[21] Braddock CH III, Fihn SD, Levinson W, Jonsen AR, Pearlman RA. How doctors and patients discuss routine clinical decisions: informed decision making in the outpatient setting. J Gen Intern Med. 1997;12(6):339-345. doi:10.1046/j.1525-1497.1997.00057.x 9192250 PMC1497128

[22] Matiasek J, Wynia MK. Reconceptualizing the informed consent process at eight innovative hospitals. Jt Comm J Qual Patient Saf. 2008;34(3):127-137. doi:10.1016/S1553-7250(08)34015-X 18419042

[23] Moris D, Shaw BI, Gloria J, . Textbook outcomes in liver transplantation. World J Surg. 2020;44(10):3470-3477. doi:10.1007/s00268-020-05625-9 32488663

[24] Karthaus EG, Lijftogt N, Busweiler LAD, ; Dutch Society of Vascular Surgery, the Steering Committee of the Dutch Surgical Aneurysm Audit, the Dutch Institute for Clinical Auditing. Textbook outcome: a composite measure for quality of elective aneurysm surgery. Ann Surg. 2017;266(5):898-904. doi:10.1097/SLA.0000000000002388 28746156

[25] Wiseman JT, Ethun CG, Cloyd JM, . Analysis of textbook outcomes among patients undergoing resection of retroperitoneal sarcoma: a multi-institutional analysis of the US Sarcoma Collaborative. J Surg Oncol. 2020;122(6):1189-1198. doi:10.1002/jso.26136 32696475

[26] Merath K, Chen Q, Bagante F, . A multi-institutional international analysis of textbook outcomes among patients undergoing curative-intent resection of intrahepatic cholangiocarcinoma. JAMA Surg. 2019;154(6):e190571. doi:10.1001/jamasurg.2019.0571 31017645 PMC6487899

[27] Griffiths EA, Hodson J, Vohra RS, ; West Midlands Research Collaborative. Utilisation of an operative difficulty grading scale for laparoscopic cholecystectomy. Surg Endosc. 2019;33(1):110-121. doi:10.1007/s00464-018-6281-2 29956029 PMC6336748

[28] Merath K, Chen Q, Bagante F, . Textbook outcomes among Medicare patients undergoing hepatopancreatic surgery. Ann Surg. 2020;271(6):1116-1123. doi:10.1097/SLA.0000000000003105 30499800

[29] Dimick JB, Birkmeyer NJ, Finks JF, . Composite measures for profiling hospitals on bariatric surgery performance. JAMA Surg. 2014;149(1):10-16. doi:10.1001/jamasurg.2013.4109 24132708 PMC4163018

[30] Dimick JB, Staiger DO, Osborne NH, Nicholas LH, Birkmeyer JD. Composite measures for rating hospital quality with major surgery. Health Serv Res. 2012;47(5):1861-1879. doi:10.1111/j.1475-6773.2012.01407.x 22985030 PMC3448279

[31] Nakazato T, Su B, Novak S, Deal SB, Kuchta K, Ujiki M. Improving attainment of the critical view of safety during laparoscopic cholecystectomy. Surg Endosc. 2020;34(9):4115-4123. Published online October 11, 2019. doi:10.1007/s00464-019-07178-y 31605213

[32] Patel MS, Hirose R, Vagefi PA. How good is good? benchmarking and the quest for quality improvement in liver transplantation. Ann Surg. 2018;267(3):426-427. doi:10.1097/SLA.0000000000002551 29319540

[33] Görgec B, Benedetti Cacciaguerra A, Lanari J, . Assessment of textbook outcome in laparoscopic and open liver surgery. JAMA Surg. 2021;156(8):e212064. doi:10.1001/jamasurg.2021.2064 34076671 PMC8173471

[34] Halpern SE, Moris D, Gloria JN, . Textbook outcome: definition and analysis of a novel quality measure in lung transplantation. Ann Surg. Published online April 29, 2021. doi:10.1097/SLA.0000000000004916 33843792

[35] van Roessel S, Mackay TM, van Dieren S, ; Dutch Pancreatic Cancer Group. Textbook outcome: nationwide analysis of a novel quality measure in pancreatic surgery. Ann Surg. 2020;271(1):155-162. doi:10.1097/SLA.0000000000003451 31274651

[36] Keller DS, Chien HL, Hashemi L, Senagore AJ, Delaney CP. The HARM score: a novel, easy measure to evaluate quality and outcomes in colorectal surgery. Ann Surg. 2014;259(6):1119-1125. doi:10.1097/SLA.0b013e3182a6f45e 24045443

[37] Daliya P, Gemmill EH, Lobo DN, Parsons SL. A systematic review of patient reported outcome measures (PROMs) and quality of life reporting in patients undergoing laparoscopic cholecystectomy. Hepatobiliary Surg Nutr. 2019;8(3):228-245. doi:10.21037/hbsn.2019.03.16 31245403 PMC6561890

